# Supplementary material for: Coenzyme-protein interactions since early life
Source: eLife. 2025 Dec 4;13:RP94174. doi: 10.7554/eLife.94174 (PMC12677900; doi:10.7554/eLife.94174)

**Supplementary File 6:** Amino acid fractional differences observed across all coenzyme binding sites. (A) Amino acid fractional difference of all coenzymes. (B) Amino acid fractional difference of all coenzymes at the residue level.


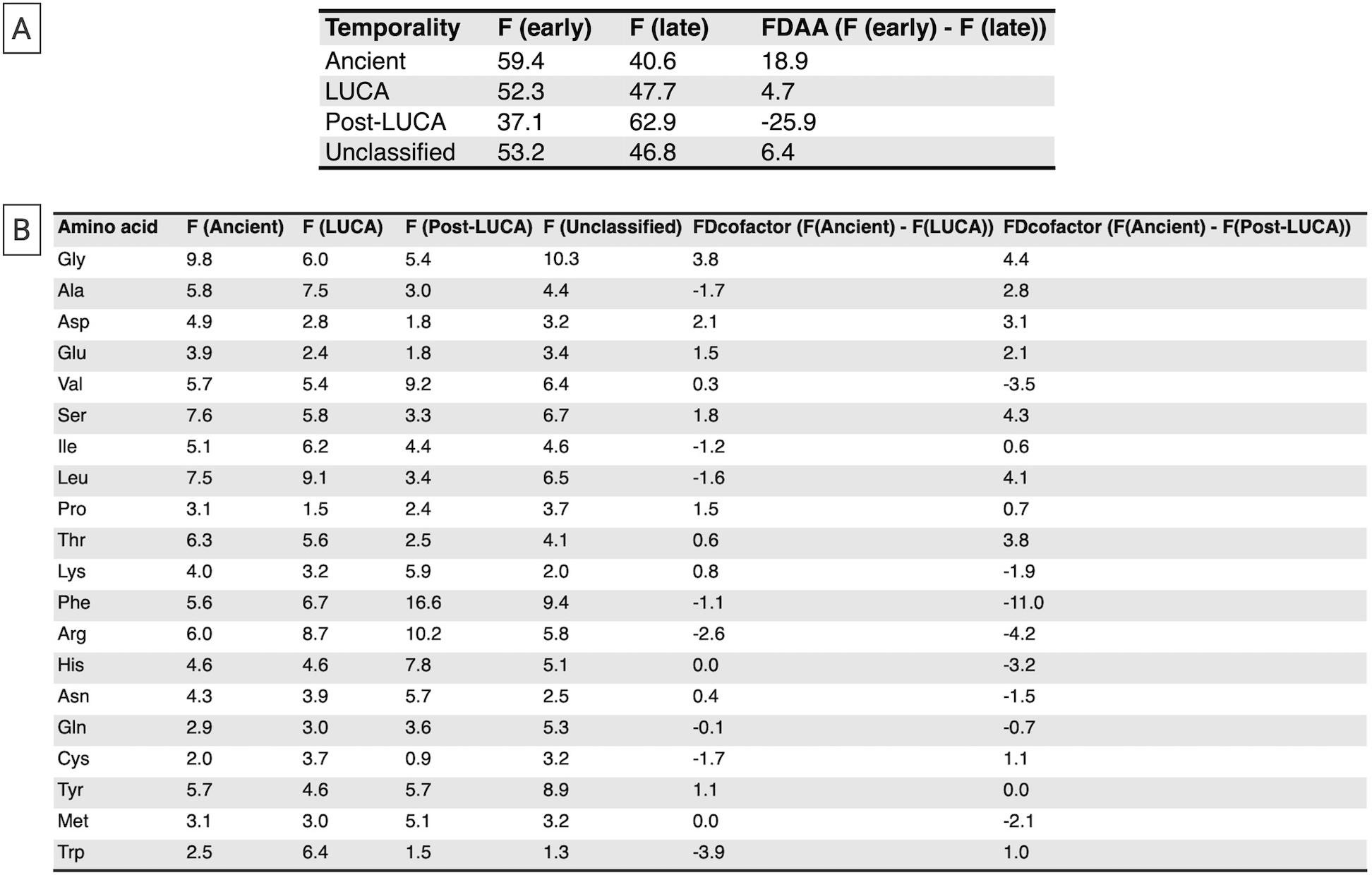

Supplement: Supplementary file 6. [file elife-94174-supp6.zip › supplementary file 6.docx]
