# Supplementary material for: Coenzyme-protein interactions since early life
Source: eLife. 2025 Dec 4;13:RP94174. doi: 10.7554/eLife.94174 (PMC12677900; doi:10.7554/eLife.94174)

**Supplementary File 7:** Amino acid fractional differences observed across all non- phosphate containing coenzyme binding sites. (A) Amino acid fractional difference of non- phosphate containing coenzymes. (B) Amino acid fractional difference of non-phosphate containing coenzymes at residue level.


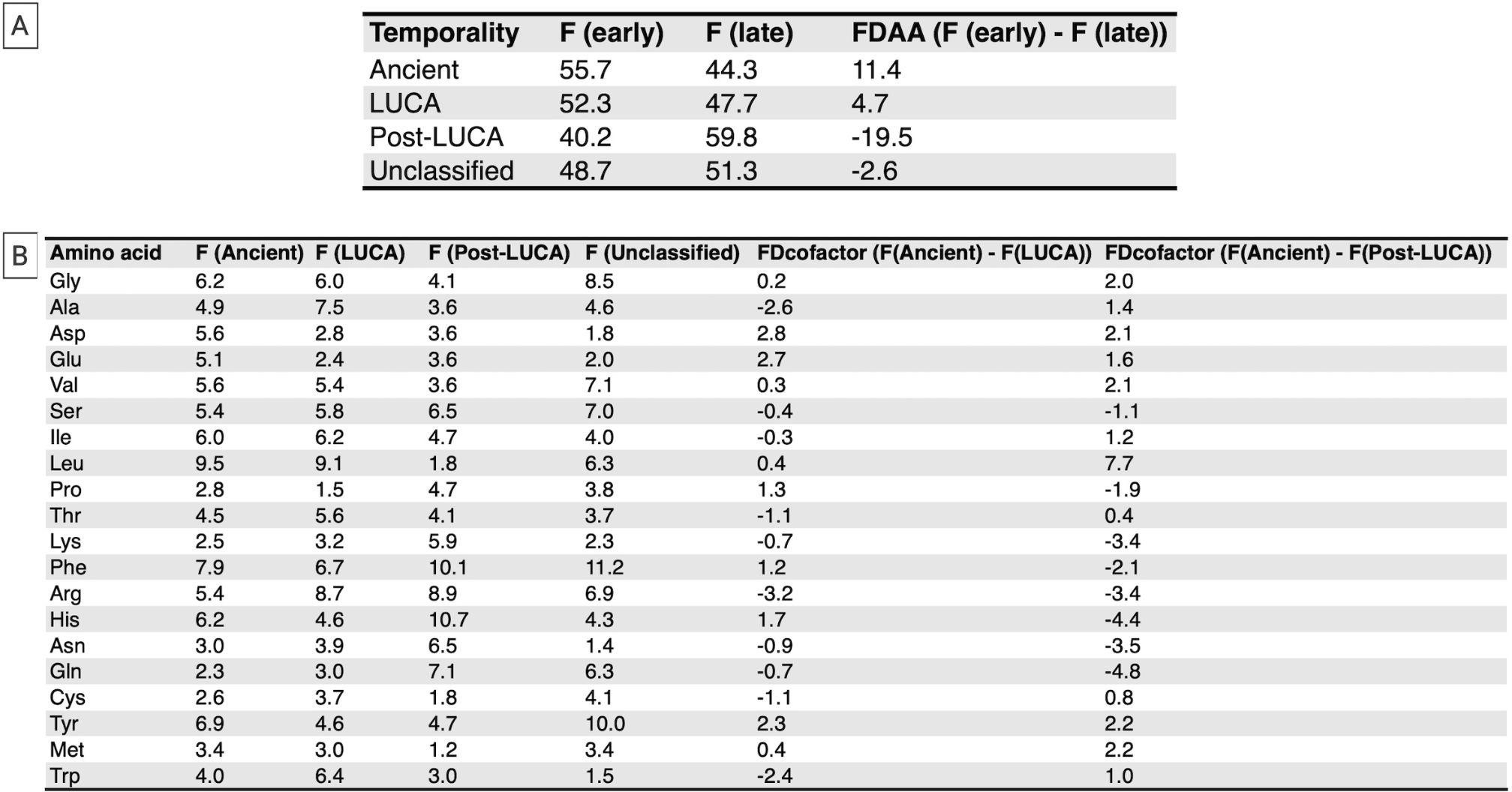

Supplement: Supplementary file 7. [file elife-94174-supp7.zip › supplementary file 7.docx]
