# Supplementary figures and images for: Coenzyme-protein interactions since early life
Source: eLife. 2025 Dec 4;13:RP94174. doi: 10.7554/eLife.94174 (PMC12677900; doi:10.7554/eLife.94174)

**Supplementary File 8:** Coenzymes interacting with nucleic acids.


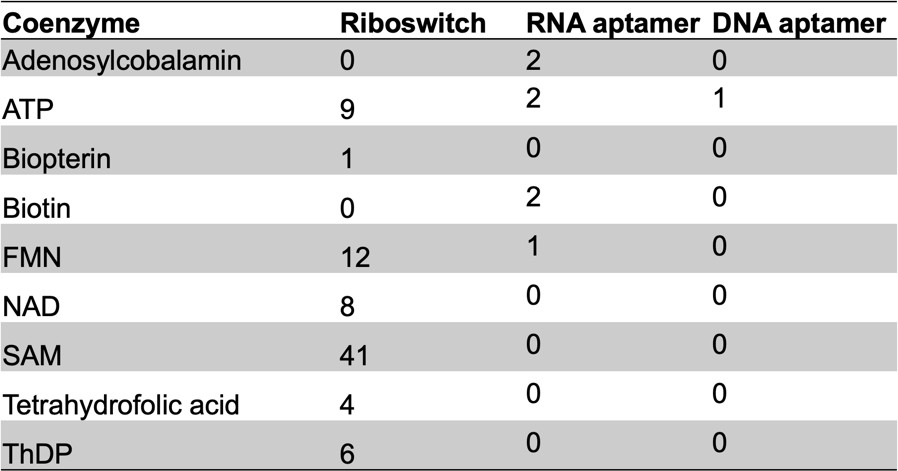

Supplement: Supplementary file 8. [file elife-94174-supp8.zip › supplementary file 8.docx]
