# Supplementary material for: Coenzyme-protein interactions since early life
Source: eLife. 2025 Dec 4;13:RP94174. doi: 10.7554/eLife.94174 (PMC12677900; doi:10.7554/eLife.94174)
Supplement: Supplementary file 9. [file elife-94174-supp9.zip › suppmlementary file 9.docx]

**Supplementary File 9:** *Chi-squared test* of early versus late amino acid composition per coenzyme class.

| Interaction type | Statistics |
| --- | --- |
| All | p-value = 0.0, chi-square = 2006.83, degrees of freedom = 26, critical value = 38.89. |
