# Supplementary material for: Coenzyme-protein interactions since early life
Source: eLife. 2025 Dec 4;13:RP94174. doi: 10.7554/eLife.94174 (PMC12677900; doi:10.7554/eLife.94174)
Supplement: Supplementary file 10. [file elife-94174-supp10.zip › supplementary file 10.docx]

**Supplementary File 10**: *Chi-squared test* comparing early versus late residue composition across all coenzyme temporalities in different interaction types.

| Interaction type | Statistics |
| --- | --- |
| Backbone | p-value = 0.01249, chi-square = 10.9, degrees of freedom = 3, critical value = 7.81 |
| Backbone & Side chain | p-value = 0.02875, chi-square = 9.04, degrees of freedom = 3, critical value = 7.81 |
| Side chain | p-value = 3.19E-26, chi-square =121.78, degrees of freedom = 3, critical value = 7.81 |
