# Supplementary material for: Coenzyme-protein interactions since early life
Source: eLife. 2025 Dec 4;13:RP94174. doi: 10.7554/eLife.94174 (PMC12677900; doi:10.7554/eLife.94174)
Supplement: Supplementary file 11. [file elife-94174-supp11.zip › supplementary file 11.docx]

**Supplementary File 11:** Average secondary structure content of the different coenzyme temporalities.

| **Coenzyme/PDB** | **Secondary structure element** | **Mean** | **Standard deviation** |
| --- | --- | --- | --- |
| PDB | beta | 15.024984 | 10.849963 |
|  | loop | 33.100027 | 12.58042 |
|  | helix | 51.874989 | 18.25092 |
| Ancient | beta | 15.024984 | 6.014811 |

|  | loop | 33.100027 | 9.852286 |
| --- | --- | --- | --- |
|  | helix | 51.874989 | 8.473325 |
| LUCA | beta | 20.037688 | 18.829335 |
|  | helix | 42.819786 | 25.661259 |
|  | loop | 37.142526 | 20.321961 |
| Post-LUCA | beta | 9.171598 | 12.970598 |
|  | helix | 46.743117 | 7.527857 |
|  | loop | 44.085285 | 5.442741 |
| Unclassified | beta | 4.218911 | 3.883016 |
|  | helix | 42.233802 | 12.173157 |
|  | loop | 53.547288 | 15.245032 |
